# Perceived Effort in Football Athletes: The Role of Achievement Goal Theory and Self-Determination Theory

**DOI:** 10.3389/fpsyg.2018.01575

**Published:** 2018-08-28

**Authors:** Diogo Monteiro, Diogo S. Teixeira, Bruno Travassos, Pedro Duarte-Mendes, João Moutão, Sérgio Machado, Luís Cid

**Affiliations:** ^1^Centro de Investigação de Desporto, Saúde e Desenvolvimento Humano – CIDESD, Vila Real, Portugal; ^2^Sport Science School of Rio Maior, Polytechnic Institute of Santarém, Rio Maior, Portugal; ^3^Faculty of Physical Education and Sport, Universidade Lusófona de Humanidades e Tecnologias, Lisbon, Portugal; ^4^Department of Sport Sciences, University of Beira Interior, Covilhã, Portugal; ^5^Department of Sport and Well Being, Polytechnic Institute of Castelo Branco, Castelo Branco, Portugal; ^6^Sport, Health and Exercise Research Unit, Polytechnic Institute of Castelo Branco, Castelo Branco, Portugal; ^7^Laboratory of Physical Activity Neuroscience, Physical Activity Sciences Post-Graduate Program, Salgado de Oliveira University, Niterói, Brazil; ^8^Laboratory of Panic and Respiration, Institute of Psychiatry of Federal University of Rio de Janeiro (IPUB/UFRJ), Rio de Janeiro, Brazil; ^9^Intercontinental Neuroscience Research Group, Rio de Janeiro, Brazil

**Keywords:** self-determination theory, achievement goal theory, motivational climate, basic psychological needs, perceived effort, football, multi-group analysis, serial mediation

## Abstract

This study examined the motivational determinants of athletes perceived effort in football considering the four-stage motivational sequence at the contextual level proposed by Hierarchical Model of Intrinsic and Extrinsic Motivation: task-involving climate, basic psychological needs, self-determined motivation (SDM), and perceived effort. Additionally, SEM multi-group analysis across different age-groups (U15, U17, U19, and U21 years) and serial mediation of basic psychological needs (BPNs) and SDM on the task-involving motivational climate and the perceived effort were also analyzed. Two independent samples of male football athletes (*N* = 403, *N* = 403), aged 13–20 years were enrolled in this study. The results support the adequacy of the structural model in explaining the perceived effort of football atheltes in all samples under analysis, and was invariant across U17, U19, and U21. However, it was not invariant across U15 and U17, U19 and U21. Furthermore, results from the serial mediation showed significant indirect effects in all samples, supporting self-determination theoretical assumptions, reinforcing the importance of BPNs satisfaction and behavioral regulation in the relation in analysis. The results show that when coaches promote a task-involving climate, the BPNs satisfaction of athletes improves. This climate will facilitate the regulation of their behaviors toward more autonomous forms of motivation, with positive outcomes in the athletes perceived effort.

## Introduction

Motivation in sports context is one of the most studied cognitive variables (Roberts, 2012), and has been highlighted in the last years as a determinant of performance in football (e.g., Álvarez et al., 2009, 2012; Quested et al., 2013). In this field, the self-determination theory (SDT; Deci and Ryan, 2000) and achievement goal theory (AGT; Nicholls, 1984) were considered the most comprehensive theoretical frameworks for understanding cognitive, behavioral, and emotional patterns associated with practitioners goals in sport contexts (Duda, 2013).

Self-determination theory explains all of the determinants of intrinsic and extrinsic motivation, considering one’s personal factors and his involvement in a social context as causes of self-determined behavior (Deci and Ryan, 2008a). According to Deci and Ryan (2000), the quality of motivation is influenced by the satisfaction of the three basic psychological needs (BPNs): autonomy (feeling of independence in which the individual regulates his own actions), competence (successful interactions with the environment), and relatedness (social connection with others). These BPNs are considered to be innate and universal, indicating that they are part of all human being’s heritage. The satisfaction of these three BPNs explain individuals’ behavior along a motivational continuum, that goes from amotivation (no intention of behavior regulation or lack of willingness to act) at one end, passing through the controlled forms of motivation (external and introjected regulation), until the most self-determined forms of motivation (identified and integrated regulation and intrinsic motivation).

Several researches in the sport context have shown that athletes who perceive that their BPNs have been met, are able to better regulate their behavior in a more self-determined motivation (SDM), thereby achieving more positive consequences, such as lower dropout rates (Sarrazin et al., 2002) and increased well-being (Jowett et al., 2017). In contrast, those who perceive that their needs have not been met or frustrated tend to regulate their behavior in a less SDM way (Duda, 2013).

Additionally, self-determined or autonomous motivation has been positively associated with cognitive, emotional, and behavioral outcomes such as enjoyment, well-being, effort, among others (e.g., Guzmán and Kingston, 2012; Pope and Wilson, 2012; Jowett et al., 2017). In SDT framework, perceived effort refers to the subject’s investment of his/her abilities in what he/she is doing and reflects the level of involvement and effort put in a given activity (McAuley et al., 1989; Pope and Wilson, 2012). This may pose as a particularly important factor when considering some of the general sport demanding characteristics, like learning processes, in-task participation, and several consequential adaptive outcomes (e.g., physiological).

Still in line with this particular behavioral outcome, higher levels of SDM have shown positive associations with perceived effort, particularly when considering the influence of intrinsic motivation (Pelletier et al., 1995; Pope and Wilson, 2012). Moreover, perceptions of BPNs have been associated with higher ratings of perceived effort, both through direct and indirect effects (Standage et al., 2005; Hein et al., 2018) and, regarding environmental characteristics, autonomy-supportive behaviors by the teacher/coach have been associated with greater effort in several sport-related contexts and intentions to maintain future sports practice (Pelletier et al., 1995; Reeve et al., 2002; Standage et al., 2006).

Another important aspect that should be considered in sport context is the motivational climate created by the coach and perceived by athletes. It refers to the environment created by the coach, based on what he or she says and does, both in training and competition (Keegan et al., 2010; Harwood et al., 2015). The coach behavior is one of the most important characteristic that influence the quality of athletes motivation, considering that they (coaches) play a key role in the promotion of a more adjusted psychological climate, recognized in literature as a particular important in the promotion of enjoyment (Quested et al., 2013), persistence in sports (Sarrazin et al., 2002), prevention of dropout (Pelletier et al., 2001), as well as perceived effort (Pope and Wilson, 2012).

Thus, AGT proposes two type of climates: (a) task-involving, where learning and personal progress are emphasized, task effort is rewarded and mistakes are part of the learning process; (b) ego-involving, where the comparison between subjects and demonstration of competence is highlighted, the result is rewarded and the mistake emphasized, giving rise to punishments (Duda, 2001; Keegan et al., 2010; Harwood et al., 2015). Previous research has revealed that a coach intervention based on a task-involving climate, comparatively to an ego-involving climate, allows the development of more challenging tasks with practitioners, encouraging harder and better motivated work, in order to achieve individual and collective goals, and with a corresponding decrease in practitioners’ dropouts rates (Keegan et al., 2010; Roberts, 2012; Harwood et al., 2015).

Taking into account the relevance with which each of these theories (SDT and AGT) has been applied to the sport context (Sarrazin et al., 2002; Duda, 2013; Appleton et al., 2016; Smith et al., 2016), researchers have integrated both theories to provide a multi-theoretical framework of sport behavior (Duda, 2013). The key element that reflects the integration of the two theories is that the motivational climate might affect the regulation of athletes’ motivations because it can facilitate/inhibit the satisfaction of their BPNs (Sarrazin et al., 2007).

According to Duda (2013), the literature that integrates SDT and AGT has been concerned with the identification of key dimensions associated with the environment created by the coach (in which coach behaviors influence motivation) as well as the motivational mechanisms through which these coach behavioral dimensions influence the way athletes think, feel, and act in the sporting context. This conceptualization (Duda, 2013) presupposes that coach-created motivational climate is multidimensional and can be more or less empowering (task-involving, autonomy supportive, and socially supportive) or dis-empowering (ego-involving and controlling). The links between these two constructs (task-involving motivational climate and autonomy supportive climate) were put in evidence in the study conducted by Appleton et al. (2016). Therefore, a more empowering climate should lead to BPN’s satisfaction, while a more dis-empowering climate should lead to the thwarting of BPNs (Duda, 2013).

Therefore, from a contextual point of view, the AGT addresses the coach-created a motivational climate and its relation to cognitions, affects, and behaviors, while SDT explains how the contextual factors influence the motivation of the subject through BPN’s satisfaction (Duda, 2013). The Hierarchical Model of Intrinsic and Extrinsic Motivation (HMIEM), was proposed by Vallerand (1997) based in SDT principles (Deci and Ryan, 1985), explaining the function of the three levels of generality: global (personality), contextual (domains of life), and situational (state). According to Vallerand (1997), the sequence starts with: social factors (where the climate created by the coach was included), followed by BPNs (autonomy, competence, and relatedness) until the types of motivation (self-determined and non-self-determined forms or autonomous and controlled forms) and consequences (perceived effort).

In line with that, the key element to combine both theories is to consider that the motivational climate might affect the athletes’ motivational regulation because it can facilitate/inhibit their BPNs satisfaction (Adie et al., 2008; Duda, 2013; Appleton et al., 2016). In fact, the literature that integrates both theories in the sport context has shown that a task-involving motivational climate positively allow BPNs (Álvarez et al., 2009, 2012; Appleton et al., 2016), while an ego-involving motivational climate had the opposite result and could lead to BPN frustration achievements (Duda, 2013; Appleton et al., 2016).

Some authors (Álvarez et al., 2012) demonstrated that satisfaction of needs is predicted by a task involving climate in football context, showing higher levels of intrinsic motivation in athletes, revealing greater intentions to remain involved in the practice, and higher levels of subjective vitality. Reinboth and Duda (2006) also show similar results, emphasizing that a climate that promotes autonomy, exhibiting the involvement for a task (Duda, 2013), has a positive and significant influence in psychological needs satisfaction during a sporting season in university athletes. Some studies have empirically tested the full-sequence postulated by Vallerand (1997). For example, Sarrazin et al. (2002) demonstrated that each of the BPN and non-SDM forms were predicted significantly and positively, being associated with higher intentions to dropout. However, according to Pope and Wilson (2012), the HMIEM needs to be analyzed in other sports and consider different competitive levels. To our knowledge, no study has analyzed the impact of the motivational climate created by the coach in satisfying BPNs, regulation motivation, and possible consequences in perceiving athletes’ effort. Thus, the main goals of this study were: (i) to test the motivational determinants of athletes perceived effort in football considering the four-stage motivational sequence at the contextual level proposed by HMIEM (Vallerand, 1997): motivational climate (task-involving climate) → BPNs → types of motivation (SDM) → consequences (perceived effort); (ii) to test the structural equation modeling (SEM) multi-groups analysis across samples and age groups (under 15 years; under 17 years; under 19 years, and under 21 years); and (iii) to analyze the mediation role of BPNs and SDM on the task-involving climate and the perceived effort.

## Materials and Methods

### Participants

Two independent samples of athletes of several football clubs from Portugal were enrolled in this study. The first sample comprised 403 football players and reflected the model calibration sample (CS); the second sample comprised 403 football players and reflected the model validation sample (VS). For multi-group analysis, the total sample was divided into different age groups (under 15 years; U15), (under 17 years; U17), (under 19 years; U19), and (Under 21 years; U21). Samples characteristics are presented in **Table 1**.

**Table 1:** Characteristics of the samples.

Samples	*N*	Age	Training experience	Weekly training sessions
CS	403	13–20	1–15	2–6
		(*M* = 16.59; *SD* = 2.23)	(*M* = 8.01; *SD* = 3.52)	(*M* = 3.68; *SD* = 0.887)
VS	403	13–20	1–15	2–6
		(*M* = 16.51; *SD* = 2.32)	(*M* = 7.78; *SD* = 3.64)	(*M* = 3.56; *SD* = 0.845)
U15	203	13–14	1–9	2–6
	(CS = 96; VS = 107)	(*M* = 13.69; *SD* = 0.462)	(*M* = 4.56; *SD* = 2.37)	(*M* = 3.27; *SD* = 0.688)
U17	206	15–16	5–10	2–6
	(CS = 99; VS = 107)	(*M* = 15.49; *SD* = 0.501)	(*M* = 7.85; *SD* = 1.66)	(*M* = 3.69; *SD* = 0.870)
U19	197	17–18	2–13	3–6
	(CS = 117; VS = 80)	(*M* = 17.41; *SD* = 0.493)	(*M* = 7.74; *SD* = 3.15)	(*M* = 3.88–0.921)
U21	200	19–20	4–15	3–6
	(CS = 92; VS = 108)	(*M* = 19.70; *SD* = 0.460)	(*M* = 11.47; *SD* = 3.11)	(*M* = 3.65; *SD* = 0.866)


CS, calibration sample; VS, validation sample; U15, under 15 years old; U17, under 17 years old; U19, under 19 years old; U21, under 21 years old; *M*, mean; *SD*, standard deviation.

### Measures

Motivational Climate Sport Youth Scale (Smith et al., 2008) – Portuguese version (MCSYSp: Monteiro et al., 2018a) was used. This questionnaire comprises eight items with a five-point Likert scale, which varied between 1 (“Strongly Disagree”) and 5 (“Strongly Agree”). The items are grouped into two factors (with four items each), which reflected the two dimensions underlying AGT framework (Nicholls, 1984). However, in this study, only the four items of the task-involving climate subscale were used.

Basic Psychological Needs Exercise Scale (Vlachoupoulos and Michailidou, 2006). The Portuguese version adapted to sports contex by Monteiro et al. (2016) was used in present study. This questionnaire encompassed 12 items with a five-point Likert scale, which varied between 1 (“Strongly Disagree”) and 5 (“Strongly Agree”). The items are grouped into three factors (with four items each), which reflected BPN’s underlying SDT (Deci and Ryan, 2000). For the purpose of this study, a second-order factor underlying the three BPNs was used (i.e., representing the composite factor of BPNs). Previous studies supported the use of second-order factor (e.g., Quested et al., 2013; Monteiro et al., 2016).

Behavioral Regulation Sport Scale (Lonsdale et al., 2008) – Portuguese version (Monteiro et al., 2018b) was used. This questionnaire included 24 items with a seven-point Likert scale, which varies between 1 (“Nothing True for Me”) and 7 (“Totally True for Me”). The items are grouped into six factors (with four items each), which reflect the motivational continuum of SDT (Deci and Ryan, 2000). For the purpose of this study, one construct was created, thus representing SDM (intrinsic motivation, integrated, and identified regulations) as suggested by Pelletier and Sarrazin (2007). Previous studies supported this methodological procedure (Álvarez et al., 2009; Clancy et al., 2017; Keshtidar and Behzadnia, 2017).

Intrinsic motivation inventory (IMI; McAuley et al., 1989) – Portuguese version (Fonseca and Brito, 2001) was used. However, for the purpose of this study, only the subscale of “Perceived Effort” was used. This sub-scale comprise five items (2, 6, 10, 14, 17) with a five-point Likert scale, which varied between 1 (“strongly disagree”) and 5 (“strongly agree”), noting that the score of 14 and 17 items was previously reversed because of its semantic formulation its semantic formulation^1^.

### Procedures

After obtaining authorization from the clubs executive board to conduct the research, all parents/legal guardians of athletes under 18 years old were contacted by the first researcher so that written informed consent was obtained, authorizing their children/athletes to participate in the research. However, for the athletes greater than or equal to 18 years old, only written informed consent was obtained. To promote honesty in the answers and guarantee the confidentiality of data, all information was collected anonymously. Before data collection, ethical approval was obtained from the committee of the Research Center in Sports Sciences, Health Sciences and Human Development (CIDESD), unit that is registered in the Portuguese National Science Foundation (FCT) under the reference UID/DTP/04045/2013. The data from the questionnaires were collected at the beginning of the training sessions in about 25 min. Twelve months after collecting data from the CS group, we collected information from VS group. This is the reason why individuals were not randomly assigned to the sample groups.

### Statistical Analysis

Descriptive statistics (means and standard deviations) as well as bivariate correlations were calculated for all variables in the two samples (CS and VS).

Although there is no consensus in the literature regarding the minimum number of subjects to perform the SEM (Barret, 2007; Hair et al., 2014), several authors suggest as a recommendation, between number of subjects and number of model parameters to be estimated, a ratio of 10:1 (advisable) or 5:1 (minimum) (Bentler and Chou, 1987; Worthington and Whittaker, 2006; Kline, 2016). Thus, in the present study, the model under analysis has 29 parameters to be estimated, therefore the recommended ratio (i.e., 10:1) was not fulfilled only in the subgroup model analysis (U15, U17, U19, and U21). In order to avoid this possible limitation, we proceeded to calculate the required sample size, according to the model under analysis (i.e., number of predictors), using GPower 3.1 software (Faul et al., 2009) with the results pointing (effect size *f*^2^ = 0.1; α = 0.05; statistical power = 0.95) that the minimum required size would be 176 subjects, which was respected in the present study.

For analyzing relationships between constructs, structural equation with maximum likelihood (ML) estimated method was performed. SEM analysis was performed according to the recommendations of several authors (Byrne, 2010; Hair et al., 2014), namely: chi-squared (χ^2^), degrees of freedom (*df*), and the level of significance (*p*), as well as, the traditionally goodness-of-fit indexes: standardized root mean square residual (SRMR), comparative fit index (CFI), root mean square error of approximation (RMSEA), and the respective confidence interval (90% CI). For these goodness-of-fit-indexes the following cut-off values suggested by several authors (Marsh et al., 2004; Byrne, 2010; Hair et al., 2014) were used: SRMR ≤ 0.08, CFI ≥ 0.90, and RMSEA ≤ 0.08; and the Cronbach’s alpha (α) to assess the reliability of the factors, considering α ≥ 0.70 as cutting values (Nunnally, 1978). Finally, confidence interval 90% and *p*-value for all structural weights were also analyzed (Hair et al., 2014). The analyses were undertaken using SPSS 20.0 and AMOS 20.0.

### Cross-Validation and SEM Multigroup Analysis

Cross-validation and SEM multigroup analysis were performed, in order to demonstrate that this model can be replicated in different groups, as suggested by Byrne (2010). These analysis were undertaken in line with previous research (Cheung and Rensvold, 2002; Byrne, 2010): (1) the structural model should be adjusted to each group; (2) a multigroup analysis was performed examining the following invariance types: unconstrained model; measurement weights; structural weights; measurement intercepts, structural residuals, and measurement residuals. Invariance assumptions were verified through the differences of the CFI with ΔCFI ≤ 0.01 (Cheung and Rensvold, 2002; Byrne, 2010). The analysis was undertaken using AMOS 20.0.

### Mediation Analysis

Considering theoretical and practical implications, serial mediation procedures were used to access mediation effects in the proposed causal model. Preacher and Hayes (2008) PROCESS macro for SPSS used grounded in the model 6 path analysis. With this procedure, the direct and indirect effects of *X* on *Y*, while modeling a process in which *X* causes M1, and, in turn, causes M2, concluding with the outcome *Y*, can be studied (Preacher and Hayes, 2008; Hayes, 2013). The proposed model (i.e., model 6) ensures the control of the indirect effects for other estimated variables, allowing also independent mediator effect analysis and regression coefficients for each causal steps of the indirect effects. Bias-corrected bootstrapped point estimates for the indirect effects of the independent variable on the dependent variable were estimated, considering standard errors and 95% confidence intervals. Significant indirect effects were considered (at alfa = 0.05) if its 95% confidence intervals does not include zero. Bias corrected and accelerated intervals supported by a 5000 samples bootstrapping were used to make inferences. Bootstrapping procedures have been recommended by MacKinnon et al. (2004) as more efficient than the normal theory approach and more powerful detecting indirect effects in smaller samples.

## Results

### Preliminary Analysis

No missing values were registered, while six cases (CS) and eight cases (VS) emerged either as univariate outliers (*z* > 3.00) and multivariate outliers (*D*^2^= *p*1 < 0.001, *p*2 < 0.001). These participants were removed to conduct further analysis. Skewness and Kurtosis values (between -2 and +2 and -7 and +7, respectively) revealed no deviations from univariate normality (Hair et al., 2014). However, the multivariate kurtosis of Mardia’s coefficient was greater than to 5.0 in all samples under analysis. Consequently, bootstrap Bollen–Stine (2000 samples) was performed (Nevitt and Hancock, 2001).

Analysis of differences between groups was also performed for the variables under analysis (i.e., task-involving climate, BPNs, SDM, and perceived effort). The one-way ANOVA revealed no differences (*p* > 0.05) between age groups (U15, U17, U19, and U21). Descriptive statistics, internal reliability scores, and bivariate correlation for all variables under analysis are presented in **Table 2**. Participants demonstrated high mean scores for all the constructs (i.e., above the midpoint).

**Table 2:** Descriptive statistics including means, standard deviations, correlations, and composite reliability for calibration and validation samples.

Factors	TI	BPN	SDM	PE	α-CS	α-VS
TI	1	0.442^∗∗^	0.437^∗∗^	0.252^∗∗^	0.66	0.66
BPN	0.798^∗∗^	1	0.574^∗∗^	0.327^∗∗^	0.84	0.85
SDM	0.666^∗∗^	0.777^∗∗^	1	0.433^∗∗^	0.86	0.88
PE	0.420^∗∗^	0.459^∗∗^	0.497^∗∗^	1	0.72	0.77
*M* ± *SD* - CS	3.01 ± 0.411	3.67 ± 0.442	4.35 ± 0.615	2.74 ± 0.389	–	–
*M* ±*SD* - VS	3.59 ± 0.402	3.30 ± 0.363	5.22 ± 0.694	2.97 ± 0.436	–	–


TI, task-involving climate; BPN, basic psychological needs; SDM, self-determined motivation; PE, perceived effort; *M*, mean; *SD*, standard deviation; CS, calibration sample; VS, validation sample; Min, minimum value; Max, maximum value; α, Cronbach’s alpha; values below the diagonal are from CS; values above the diagonal are from VS. ^∗∗^*p* < 0.001.

As we can see, **Table 3** shows that all structural models adjusted to the data according to cut-off values adopted in the methodology, except for the SRMR in the U17 and U19 samples.

**Table 3:** Goodness-of-fit indexes for all structural models.

Models	χ^2^	*df*	B-S*p*	SRMR	CFI	RMSEA	90% CI
Model 1 (CS)	198.090	62	<0.001	0.074	0.911	0.073	0.062–0.085
Model 1 (VS)	161.419	62	<0.001	0.066	0.943	0.063	0.051–0.075
U15	136.181	62	<0.001	0.055	0.922	0.077	0.059–0.095
U17	134.859	62	0.002	0.097	0.907	0.076	0.058–0.093
U19	137.240	62	<0.001	0.081	0.920	0.079	0.061–0.096
U21	121.405	62	0.004	0.074	0.919	0.069	0.051–0.088


CS, calibration sample; VS, validation sample; SM, structural models χ^2^, Chi-square; *df*, degrees of freedom; B–S*p*, Bollen–Stine bootstrap level of significance (2000 samples); SRMR, standardized root mean square residual; CFI, comparative fit index; RMSEA, root mean squared error of approximation; 90% CI, confidence interval of RMSEA.

**FIGURE 1:**
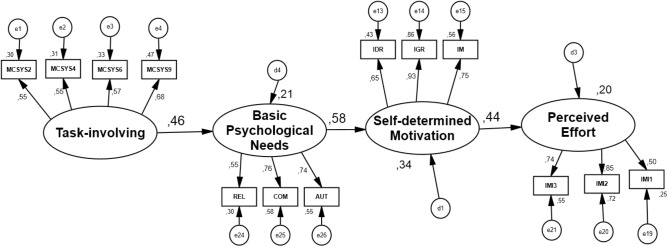
Individual standardized parameters of the initial hypothetical model (validation sample). MCSYS from 2 to 9 representing the items of the scale. COM, competence; AUT, autonomy; REL, relatedness; IDR, identified regulation; IGR, integrated regulation; IM, intrinsic motivation; perceived effort, from 1 to 3 representing the items of the scale; E, measurement errors of each of the items and factor.

As we can observe in **Figures 1** (CS), **2**), there are a positive and significant effects among all constructs. For CS it was observed that: task-involving – BPNs (β = 0.46; 90% CI 0.333–0.594, *p* = 0.001); BPNs – SDM (β = 0.58; 90% CI 0.469–0.683, *p* = 0.001); SDM-perceived effort (β = 0.44; 90% CI 0.344–0.540, *p* = 0.001). Standardized indirect effects also showed a positive and significant effect between task-involving climate and SDM (β = 0.27; 90% CI 0.166–0.392, *p* = 0.001) through BPNs. Also, task-involving predicted perceived effort (β = 0.12; 90% CI 0.070–0.197, *p* = 0.001), through SDM, and BPNs predicted perceived effort (β = 0.28; 90% CI 0.178–0.346, *p* = 0.001) through SDM. For VS it was observed that: task-involving – BPNs (β = 0.67; 90% CI 0.576–0.764, *p* = 0.001); BPNs – SDM (β = 0.68; 90% CI 0.589–0.762, *p* = 0.002); SDM – perceived effort (β = 0.43; 90% CI 0.321–0.529, *p* = 0.001). Standardized indirect effects also showed a positive and significant effect between task-involving climate and SDM (β = 0.46; 90% CI 0.321–0.529, *p* = 0.001), through BPNs, as well as between task-involving climate and perceived effort (β = 0.19; 90% CI 0.129–0.281, *p* = 0.001) through SDM, and BPNs predicted perceived effort (β = 0.30; 90% CI 0.203–0.390, *p* = 0.001) through SDM.

**FIGURE 2:**
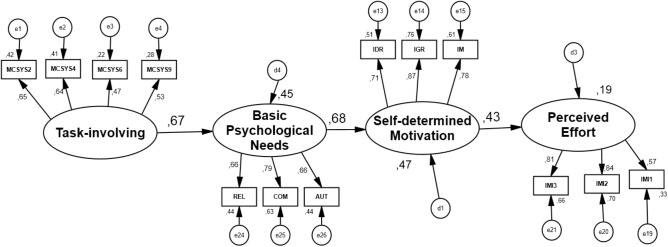
Individual standardized parameters of the initial hypothetical model (calibration sample). MCSYS from 2 to 9 representing the items of the scale. COM, competence; AUT, autonomy; REL, relatedness; IDR, identified regulation; IGR, integrated regulation; IM, intrinsic motivation; perceived effort, from 1 to 3 representing the items of the scale; E, measurement errors of each of the items and factor.

The direct and indirect effects among all constructs in samples under analysis are provided in **Table 4**. In general, results revealed a positive and significant effect from both direct and indirect paths, except for the U21 sample (i.e., SDM–PE). In the U15 sample, due to a BPNs – self-determined motivation relation superior to 0.84 (Hair et al., 2014) (i.e., β = 0.91), a possible multicollinearity issue could be present. For this matter, and accordingly with some authors recommendations (Hair et al., 2014), the variance inflation factor (VIF) was calculated, assuming < 3.00 as cut-off value, in order to discard multicollinearity issues. The VIF for these variables was 1, rejecting that possibility (Hair et al., 2014).

**Table 4:** Direct and indirect effects analysis among all constructs.

Path	TI→BPN	TI→SDM	TI→PE	BPN→SDM	BPN→PE	SDM→PE
U15	β = 0.79	β = 0.71	β = 0.53	β = 0.91	β = 0.67	β = 0.74
	(90% CI = 0.621–0.879), *p* = 0.002	(90% CI = 0.513–0.842), *p* = 0.002	(90% CI = 0.364–0.664), *p* = 0.001	(90% CI = 0.792–0.986), *p* = 0.001	(90% CI = 0.532–0.792), *p* = 0.001	(90% CI = 0.630–0.841), *p* = 0.001
U17	β = 0.42	β = 0.24	β = 0.09	β = 0.58	β = 0.22	β = 0.37
	(90% CI = 0.252–0.587), *p* = 0.001	(90% CI = 0.132–0.388), *p* = 0.001	(90% CI = 0.041–0.171), *p* = 0.001	(90% CI = 0.450–0.701), *p* = 0.001	(90% CI = 0.127–0.334), *p* = 0.001	(90% CI = 0.228–0.510), *p* = 0.001
U19	β = 0.57	β = 0.28	β = 0.11	β = 0.49	β = 0.20	β = 0.41
	(90% CI = 0.428–0.714), *p* = 0.001	(90% CI = 0.162–0.425), *p* = 0.001	(90% CI = 0.056–0.221), *p* = <0.001	(90% CI = 0.331–0.618), *p* = 0.001	(90% CI = 0.110–0.331), *p* = 0.001	(90% CI = 0.263–0.563), *p* = 0.001
U21	β = 0.49	β = 0.27	β = 0.05	β = 0.55	β = 0.09	β = 0.17
	(90% CI = 0.314–0.678), *p* = < 0.001	(90% CI = 0.145–0.455), *p* = <0.001	(90% CI = 0.008–0.115), *p* = 0.043	(90% CI = 0.380–0.716), *p* = 0.001	(90% CI = 0.007–0.207), *p* = 0.008	(90% CI = 0.010–0.326), *p* = 0.084


TI, task-involving; BPN, basic psychological needs; SDM, self-determined motivation; PE, perceived effort.

With respect to structural model invariance (**Tables 5, 6**), the results support the structural equivalence across samples (calibration and validation) and across U17, U19, and U21 age groups. Therefore, all factor loadings, structural paths, factor covariances, factor residual variances, and measurement error variances are operating equivalently across samples, as well as across age groups (ΔCFI < 0.01). However, the results did not show evidence of invariance across U15 and remaining groups (ΔCFI > 0.01).

**Table 5:** Goodness-of-fit indexes for the invariance of the structural model across samples and age-groups.

Models	χ^2^	*df*	Δχ^2^	Δ*df*	*p*	CFI	ΔCFI
**AC–AV**							
UM	359.509	124	–	–	–	0.928	–
MW	373.822	133	14.313	9	0.112	0.926	0.002
SM	379.575	136	20.066	12	0.066	0.925	0.003
MI	380.567	137	21.058	13	0.072	0.925	0.003
SR	386.678	140	27.170	16	0.040	0.924	0.004
MR	394.953	153	35.444	29	0.190	0.926	0.002
**U15–U17**							
UM	276.355	124	–	–	–	0.913	–
MW	298.160	133	21.806	9	0.010	0.905	0.008
SM	319.418	136	43.063	12	<0.001	0.895	0.018
MI	325.839	137	49.484	13	<0.001	0.892	0.021
SR	330.391	140	54.037	16	<0.001	0.891	0.022
MR	379.727	153	103.373	29	<0.001	0.870	0.043
**U15–U19**							
UM	273.421	124	–	–	–	0.921	–
MW	283.904	133	10.483	9	0.313	0.920	0.001
SM	307.380	136	33.959	12	0.001	0.909	0.012
MI	315.092	137	41.671	13	<0.001	0.906	0.015
SR	327.142	140	53.721	16	<0.001	0.901	0.020
MR	427.408	153	153.987	29	<0.001	0.855	0.066
**U15–U21**							
UM	257.328	124	–	–	–	0.921	–
MW	275.582	133	18.254	9	0.032	0.915	0.006
SM	305.391	136	48.062	12	<0.001	0.899	0.022
MI	317.036	137	59.707	13	<0.001	0.893	0.028
SR	325.620	140	68.292	16	<0.001	0.890	0.031
MR	401.389	153	144.061	29	<0.001	0.852	0.069


CS, calibration sample; VS, validation sample; χ^2^, Chi-square; Δχ^2^, differences in value of chi-square; Δ*df*, differences in degrees of freedom; *p*, level of significance; CFI, comparative fit index; ΔCFI, differences in the value of the comparative fit index; UM, unconstrained model; MW, measurement weights; SM, structural weights; MI, measurement intercepts; SR, structural residuals; MR, measurement residuals.

**Table 6:** Goodness-of-fit indexes for the invariance of the structural model across age-groups.

Models	χ^2^	*df*	Δχ^2^	Δ*df*	*p*	CFI	ΔCFI
**U17–U19**							
UM	277.415	124	–	–	–	0.912	–
MW	285.816	133	8.401	9	0.494	0.912	0.000
SM	290.778	136	13.364	12	0.343	0.911	0.001
MI	290.870	137	13.455	13	0.413	0.911	0.001
SR	294.321	140	16.907	16	0.392	0.911	0.001
MR	313.199	153	35.784	29	0.180	0.908	0.004
**U17–U21**							
UM	261.320	124	–	–	–	0.910	–
MW	273.061	133	11.740	9	0.228	0.908	0.002
SM	276.836	136	15.516	12	0.214	0.908	0.002
MI	277.193	137	15.873	13	0.256	0.908	0.002
SR	278.785	140	17.465	16	0.356	0.909	0.001
MR	306.727	153	45.407	29	0.027	0.899	0.011
**U19–U21**							
UM	258.389	124	–	–	–	0.920	–
MW	270.711	133	12.322	9	0.196	0.918	0.002
SM	278.814	136	20.426	12	0.059	0.915	0.005
MI	279.287	137	20.899	13	0.075	0.915	0.005
SR	281.768	140	23.380	16	0.104	0.915	0.005
MR	305.623	153	47.235	29	0.018	0.909	0.011


χ^2^, Chi-square; Δχ^2^, differences in value of chi-square; Δ*df*, differences in degrees of freedom; *p*, level of significance; CFI, comparative fit index; ΔCFI, differences in the value of the comparative fit index; UM, unconstrained model; MW, measurement weights; SM, structural weights; MI, measurement intercepts; SR, structural residuals; MR, measurement residuals.

**FIGURE 3:**
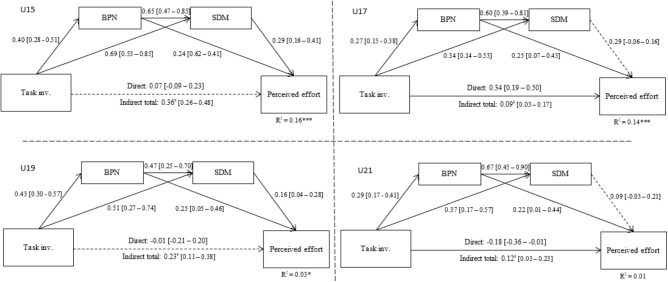
Serial mediation models for task involvement, basic psychological needs, autonomous regulations, and perceived effort. Attached file “Mediation_Models.” ^∗^*p* < 0.05; ^∗∗^*p* < 0.01; ^∗∗∗^*p* < 0.001; ¥ = 90% CI of the bias and corrected and accelerated estimate indicate a significant indirect effect; significant effect → ; non-significant effect → ; BPN, basic psychological needs; SDM, self-determined motivation.

In **Figure 3** presented are the mediating effects of BPNs and SDM in the relationship between task-involving climate and perceived effort in the four samples. Analysis shows that in all samples there are significant indirect effects, supporting SDT theoretical assumptions in the context of this study.

Task-involving climate presented in all mediation models revealed positive significant associations with BPN and SDM. BPN sustained in all groups a positive association with perceived effort, while SDM was only positively associated with perceived effort in the U15 and U19 samples. In direct effect analysis, the U17 and U21 samples showed that the task involvement was, respectively, positively (0.34, *p* < 0.001) and negatively (-0.18, *p* = 0.04) associated with perceived effort. Models predictions also seem to decrease across age: 16% (*p* < 0.001) in the U15; 14% (*p* < 0.001) in the U17; 3% (*p* < 0.05) in the U19; and no statistical significance in the U21 (1%, *p* = 0.41).

## Discussion

The aim of this study was to examine the motivational determinants of athletes perceived effort in football considering the four-stage motivational sequence at the contextual level proposed by HMIEM: task-involving climate, BPNs, SDM, and perceived effort. Additionally, SEM multi-group analysis across different age groups (U15, U17, U19, and U21 years) and the serial mediation of BPNs and SDM on the task-involving climate and the perceived effort were also analyzed.

Results of the present study show that the subjects value all the constructs underlying the hypothesized model. This is in line with previous studies, not only in football (Álvarez et al., 2012), but also in other sports (Pelletier et al., 2001; Sarrazin et al., 2002; Guzmán and Kingston, 2012; Pope and Wilson, 2012), which reveals the importance of the theoretical constructs underlying the AGT (task-involving) and SDT (BPNs and behavioral regulation) in the sports context.

It is possible yet to see that there are positive and significant bivariate correlations among all constructs under analysis (i.e., task-involving motivational climate, BPNs, SDM, and perceived effort). These evidences corroborate several studies that have been carried out with these theoretical models in several sports, such as: swimming (Pelletier et al., 2001), football (Álvarez et al., 2012), rugby (Pope and Wilson, 2012), as well as across different individual and team sports (Guzmán and Kingston, 2012).

As expected, the findings of the present study provided overall support for this model. The results show that it was adjusted to the data, in all age groups under analysis (Marsh et al., 2004; Byrne, 2010; Hair et al., 2014). In general the results show that a task-involving motivational climate is a positive and significant predictor of BPNs in all age groups. In turn, BPNs are a positive and significant predictor of SDM, and SDM is a positive and significant predictor of perceived effort. These results are justified both from a theoretical (Deci and Ryan, 2000) and an empirical point of view (Sarrazin et al., 2002; Álvarez et al., 2012; Pope and Wilson, 2012).

From a theoretical point of view, motivation is not directly related to the social factors, but through the satisfaction of the three BPNs (responsible for continuous growth, integrity, and well-being), that are the main promoters of how subjects will regulate their behavior (Deci and Ryan, 2008b). According to Duda (2013), AGT and SDT conceptualize key aspects of motivation at the contextual level, because both theories suggest that atmosphere that are less evaluative, give more support to the intrinsic desire to learn, and promote the basis to increase achievement. This reinforces the issue pointed by Duda and Appleton (2016), where the task-involving motivational climate is a climate that is autonomy supportive, that promotes empowering environment. Thus, a motivational climate, created by the coach, that promotes task involving, favors BPN’s satisfaction, being the basis of SDM (Ryan and Deci, 2007), which can be particularly important in the sport’s field, since the SDM (or autonomous motivation) is among the most important factors in behavior maintenance over time (Jõseaar et al., 2011, 2012). From an empirical point of view, these results corroborate several studies carried out in last years (Pelletier et al., 2001; Sarrazin et al., 2002; Álvarez et al., 2009, 2012; Guzmán and Kingston, 2012; Pope and Wilson, 2012; Duda, 2013), who have shown that athletes who perceive a task-involving climate feel their BPNs more satisfied, and in turn, promote high levels of SDM or autonomous motivation. The results of the present study also reinforce that task-involving motivational climate could be supportive of autonomy. According to Duda (2013) conceptualization, the coach-created a motivational climate is multidimensional and can be empowering (task-involving, autonomy supportive), since both promote BPN’s satisfaction, and also positive outcomes (e.g., enjoyment or self-worth) (Appleton and Duda, 2016). This issue could be particularly important because task-involving motivational climate can also be influenced by the ratio of autonomy and controlling coaching emphasis, since both of them can coexist in sport domain (Appleton et al., 2016).

For example, across-cultural study conducted by Jowett et al. (2017), the authors demonstrated not only the relations between a positive motivational climate, BPNs, behavior regulation, and well-being, but also that relationships remain the same in different cultures (British, Chinese, Greek, Spanish, and Swedish), which empirically demonstrates the universality of these constructs. This means that SDT constructs are universal in their importance and their effects (Deci and Ryan, 2008a).

Finally, regarding the associations between SDM and perceived effort of athletes, results demonstrate a positive and significant association in all age groups, except, in the U21. This result corroborates the evidence found by Pope and Wilson (2012), which demonstrates that autonomous motivation was a positive and significant predictor of perceived effort, which was explained by 21% (similar to our results in both samples). Thus, these results demonstrate that with the development of the sports career, the impact of autonomous motivation on perceived effort seems to lose importance.

As said above, the effect of SDM and perceived effort in U21 sample was not significant. This result reveals that SDM in this age group may not be an important predictor of perceived effort. This result contradicts some studies (e.g., Pope and Wilson, 2012), where the authors demonstrated positive and significant relationships between the SDM and perceived effort. In this sense, the perceived effort in this age group seems to be explained by other factors than those analyzed in the present study.

According to the long-term development model, the U21 group is designed as the stage – training to win. It means players at that moment of career development are focused on individual and team performance (Balyi et al., 2013). Due to the constant competition to improve their capabilities, as well as to ensure a position on the team, we can speculate that players need to maintain their focus on the efforts to train and compete, rather than on the issues related with the SDM. More than that, it is well reported that this is a critical moment of career development of players (i.e., transition from junior to senior players) and the expectations to ensure a position on the team or a contract on the club (Stambulova, 2016) could change the motivational regulation of players, emphasizing the perceived effort to improve performance. Also, the analysis of athlete–coach relationships across the career stages of players revealed that the friendliness and the emotional relations developed at the beginning of players’ career decreased to more neutral relationships, focused on results and on the work developed (Sandström et al., 2016).

Further studies should be developed to evaluate the association between BPNs, SDM, and perceived effort in different levels of career development and in different levels of performance in order to substantiate these findings.

With regard to the structural invariance tests, our results showed that the model was structurally invariant across samples and age groups (U17, U19, and U21), thereby confirming the equivalence of the model across different groups of players. This fact reveals the importance of these relationships in the perceived effort of the athletes, thus demonstrating the suitability of this model in the specific context of football.

Referring to U15, structural invariance assumptions were not verified (ΔCFI > 0.01), which shows that neither the effects observed between the variables, nor the respective theoretical assumptions under analysis, can be interpreted between U15 and the remaining age groups (Byrne, 2010). According to Chen (2008) and Sass (2011), it is not legitimate to compare results between U15 and other age groups, since any result obtained may be biased since the model did not reveal invariance criteria. This seems to be linked to the specific characteristic of this age group. According to Sandström et al. (2016), there is an increase in friendliness between the initiation and the following levels of sport/practice development, and a decrease in more advanced stages, showing a more neutral emotional relationship. Coaches’ involvement was higher on the development stages, and changed gradually on later stages, according to athletes’ perceptions.

As for the mediation effects, in addition to the theoretical postulates advocated by Deci and Ryan (2000, 2008a) and Ryan and Deci (2007) in SDT, as well as for HMIEM proposed by Vallerand (1997) and empirically confirmed in several studies in the sports context (e.g., Álvarez et al., 2012; Jowett et al., 2017), there are also relevant issues from a statistical point of view, since the observed indirect effects between the variables are significant, which according to Hair et al. (2014), is suggestive of mediation, which reinforces the pertinence of the analysis made.

The mediation analysis provided additional information that partially supports previous interpretations. In all samples there were significant indirect effects, supporting theoretical SDT (Deci and Ryan, 2000, 2008a) and HMIEM (Vallerand, 1997) assumptions, reinforcing the importance of BPN’s satisfaction and behavioral regulation in the relation between task-involving climate and perceived effort in football. In the present sample, the mediating effects, direct effects (except in U17), and prediction values seem to decrease across age (i.e., more specifically, sport age groups), showing a tendency that anticipates a shift of the influence of the task-involving climate on the perceived effort in this sport activity across age groups. This may reflect that across age groups, and with a sport specialization associated with each phase of the athlete development, the climate perception is changed.

Considering that in the latter two age samples (U19 and U21) there is a phase of transition to a more specialized and, in some degree, more professional approach to the training and competition processes, classified as “advanced training” by FIFA (n.d.). It is possible to assume that the inevitable search and inter-individual competition for the performance and results in this advanced training phase may lead to a secondary position in the team play and/or draft, which in turn could possibly be interpreted by the athletes as a form of punishment by the coach (Keegan et al., 2010, 2014).

In fact, the task-involving climate and its characteristics were learning and personal progress are accepted, effort is rewarded and mistakes are considered as a normal part of the learning process, are very similar to the sports pedagogy approaches commonly used in youths training (e.g., Cassidy et al., 2004), and a shift in some coaching process are expected through more specialized approaches.

Additionally, SDT mediators presented a similar tendency as previous causal steps analysis. The BPN’s satisfaction and SDM influence are particularly evident in the U15 and U17 age groups, being in line with some similar studies (e.g., Álvarez et al., 2009; Almargo et al., 2015), with the exception of the SDM in the U17 sample. In the serial mediation analysis, BPNs stand out as particularly important in the understating of these relations, because of the (i) positive association with the task-involving climate, (ii) positive association with perceived effort (and expectedly, with many other behavioral, cognitive, and affective outcomes that are not the main focus of this study, but reflect important aspects in physical activity/sports contexts; for review Ng et al., 2012; Teixeira et al., 2012), and (iii) positive association with autonomic regulations, that, despite not significant with perceived effort in the U17 and U21 samples, are reported in the literature as important to obtain better behavioral outcomes (Deci and Ryan, 2008a,b; Almargo et al., 2015).

These results tend to increasingly support the importance of BPN in sport context and particularly in youths football. Despite being in an early phase of study about possible interactions of all these variables (task-involving climate, BPN, behavioral regulation, and perceived effort interactions across age groups, and levels of participation/competition), the present study contributed to the dissemination of knowledge in the context of football, and corroborates the assumptions of Ryan (1995), which states that the research carried out with SDT should be done in a specific context.

However, although the present study demonstrates contextual and motivational determinants of perceived effort in football, some limitations should be accounted, in order to better understand the possible implications of present findings. Therefore, it should be considered that (i) the present study is of a cross-sectional nature, so we advised that future studies should analyze the same variables and methodology in a longitudinal/experimental way; (ii) future studies are encouraged to complement the measure of perceived effort with other observable physiological indicators (e.g., heart rate variability, lactate and cortisol measures) that may unveil theoretically expected relations between psychological perceived effort (i.e., behavioral consequence; reported levels of effort in the activity) and physiological adaptations; (iii) future studies should make an effort to analyze the effect of each BPN and needs frustration, in this particular context, because youth football athletes may perceive the characteristics of each level of practice differently, due to the inherent characteristic of training/competition process. Additionally, motivational regulation analysis (consequences of BPN’s satisfaction/frustration and motivational climate) in athletes performance should also be addressed in future studies; (iv) taking into account the multidimensional approach to the coach-created a motivational climate (Duda, 2013; Appleton and Duda, 2016; Duda and Appleton, 2016), that integrates the major social environmental dimensions emphasized within AGT and SDT, future studies should analyze the combined effect of the task-involving climate and autonomy supportive climate (i.e., empowering motivational climate), on the BPNs, motivational regulation, and different behavioral, emotional, and cognitive outcomes, in the context of football, as in other sports contexts; (v) ability heterogeneity in groups may influence competence perceptions, and may justify future ability profile analysis; (vi) taking into account the assumptions of HMIEM, the present study was conducted at the contextual level. However, situational motivation is also important to self-regulatory processes such as goal confidence, goal setting, and affect, which may operate in a cyclical manner. For that reason, future studies may address these issues to further understand the aforementioned relations.

Nevertheless, from the results of the present study we can draw some implications for the practice: (i) coaches that promote a task-involving-climate and improve the BPN’s satisfaction of athletes. This climate will facilitate the regulation of their behaviors toward more autonomous forms of motivation, with positive results in athletes’ perceived effort. Moreover, when coach-created a task-involving motivational climate, he is also implicitly promoting an autonomy supportive climate (i.e., empowering motivational climate), and more positive outcomes from his athletes can be expected (e.g., more enjoyment); (ii) training planning should promote more BPN’s satisfaction, creating training dynamics that promotes the feeling of autonomy and competence and strengthen the relationship among teammates; (iii) promote evaluation of the way that athletes regulate their motivation at the beginning of the season. The quality of motivation identification will allow coaches to promote more adapted tasks and roles to each player, helping them to achieve higher levels of perceived effort.

## Author Contributions

LC, DM, JM, PD-M, BT, and DT participated in study design, data collection, and writing of the first draft manuscript. LC, DM, DT, PD-M, and SM participated in data analysis and writing of the methodology and results. DT, DM, JM, PD-M, and BT participated in data collection and in final revisions of the manuscript. All authors have read and approved the final version of the manuscript, and agree with the order of presentation of the authors.

## Conflict of Interest Statement

The authors declare that the research was conducted in the absence of any commercial or financial relationships that could be construed as a potential conflict of interest.

## Footnotes

1 The measurement model with five items presented problems with its validity, because of that in the present study was used only three items. Previous studies (e.g., Monteiro et al., 2014) founded similar problems. In Pope and Wilson (2012) study, the authors used four items, since one item: “It is important to me to do well at this task”) was removed from the original IMI item pool because the item content represented personal importance as opposed to perceived effort expended in the activity under scrutiny” (p. 96)
